# The activity of disease-causative STING variants can be suppressed by wild-type STING through heterocomplex formation

**DOI:** 10.3389/fcell.2022.1037999

**Published:** 2022-11-03

**Authors:** Ruri Shindo, Yoshihiko Kuchitsu, Kojiro Mukai, Tomohiko Taguchi

**Affiliations:** Laboratory of Organelle Pathophysiology, Department of Integrative Life Sciences, Graduate School of Life Sciences, Tohoku University, Sendai, Japan

**Keywords:** STING, STING-associated vasculopathy with onset in infancy (SAVI), membrane traffic, interferonopathy, endoplasmic reticulum (ER)

## Abstract

Stimulator of interferon genes (STING) is essential for the type I interferon response induced by microbial DNA from viruses or self-DNA from mitochondria/nuclei. Recently, gain-of-function mutations in STING have been identified in patients with STING-associated vasculopathy with onset in infancy (SAVI). The SAVI patients exhibit complex systemic vascular inflammation and interstitial lung disease, resulting in pulmonary fibrosis and respiratory failure. SAVI mouse models have recently developed, harbouring common SAVI mutations, such as N153S and V154M, which correspond to the human N154S and V155M, respectively. Interestingly, crosses of heterozygous SAVI mice did not yield homozygous SAVI mice as of embryonic day 14, indicating that homozygous SAVI embryos were not viable and that wild-type (WT) allele would function dominantly over SAVI alleles in terms of viability. However, the molecular mechanism underlying the dominance has not been understood. In the present study, we show that STING (WT) and STING (SAVI) can form heterocomplex. The heterocomplex localized primarily in the endoplasmic reticulum (ER) and failed to reach the trans-Golgi network (TGN), where STING activates the downstream kinase TBK1. SURF4 is the essential protein functioning in the retrieval of STING from the Golgi to the ER. The amount of SURF4 bound to STING (SAVI) significantly increased in the presence of STING (WT). These results suggest that STING (WT) can suppress the activity of STING (SAVI) by tethering STING (SAVI) to the ER through heterocomplex formation. The dormant heterocomplex formation may underlie, at least in part, the dominance of STING WT allele over SAVI alleles in the STING-triggered inflammatory response.

## Introduction

The cGAS-STING pathway is essential for the type I interferon response upon the emergence of cytosolic DNA (Barber, 2015; Hopfner and Hornung, 2020). Cyclic GMP-AMP (cGAMP) synthase (cGAS) (Sun et al., 2013) directly binds to the cytosolic DNA and the enzymatic activity is upregulated by the binding. Activated cGAS generates cyclic GMP-AMP (cGAMP) (Wu et al., 2013) from ATP and GTP. An ER-localized transmembrane protein STING (Ishikawa and Barber, 2008) (also known as MITA (Zhong et al., 2008), ERIS (Sun et al., 2009), MPYS (Jin et al., 2008), or TMEM173) binds to cGAMP. cGAMP-bound STING translocates to the Golgi, and activates TANK-binding kinase 1 (TBK1) at the TGN (Ishikawa and Barber, 2008; Zhong et al., 2008; Ishikawa et al., 2009; Saitoh et al., 2009; Sun et al., 2009; Mukai et al., 2016; Ogawa et al., 2018; Zhang et al., 2019; Zhao et al., 2019; Kemmoku et al., 2022). Activated TBK1 phosphorylates STING at Ser365, generating a docking site for interferon regulatory factor 3 (IRF3) (Liu et al., 2015). IRF3 recruited to STING is then phosphorylated by TBK1, and activated IRF3 translocates into the nucleus, driving the type I interferon production (Tanaka and Chen, 2012; Liu et al., 2015).

STING-associated vasculopathy with onset in infancy (SAVI) is a disorder involving abnormal inflammation throughout the body, especially in the skin, blood vessels, and lungs (Jeremiah et al., 2014; Liu et al., 2014). A number of STING variants (H72N, F153V, V147L, N154S, V155M, G158A, G166E, C206Y, G207E, R281Q/W, and R284G/S) have been identified in the SAVI patients (Jeremiah et al., 2014; Liu et al., 2014; König et al., 2017; Melki et al., 2017; Konno et al., 2018; Saldanha et al., 2018; Keskitalo et al., 2019; Lin et al., 2020; Lin et al., 2021). These disease-causative variants constitutively activate the type I interferon response without cGAMP (Jeremiah et al., 2014; Liu et al., 2014; Dobbs et al., 2015; Mukai et al., 2016). Recently, SAVI mouse models with mutations (N153S and V154M, which correspond to human N154S and V155M, respectively) have been generated (Warner et al., 2017; Motwani et al., 2019). The crosses between heterozygous mice did not yield homozygous SAVI mice, indicating that homozygous SAVI embryos were lethal and that WT allele would function dominantly over SAVI variants in terms of viability (Warner et al., 2017; Motwani et al., 2019). However, the molecular mechanism underlying its dominance has not been understood.

In the present study, we showed that STING (SAVI) can form a heterocomplex with STING (WT). The heterocomplex, not homocomplex composed of STING (SAVI), was predominantly localized to the ER and dormant. We propose that the dormant heterocomplex formation between STING (WT) and STING (SAVI) underlies, at least in part, the dominance of STING WT allele over SAVI alleles in the STING-triggered inflammatory response.

## Results

### The expression of STING (WT) suppresses the SAVI-dependent STING signalling

**FIGURE 1:**
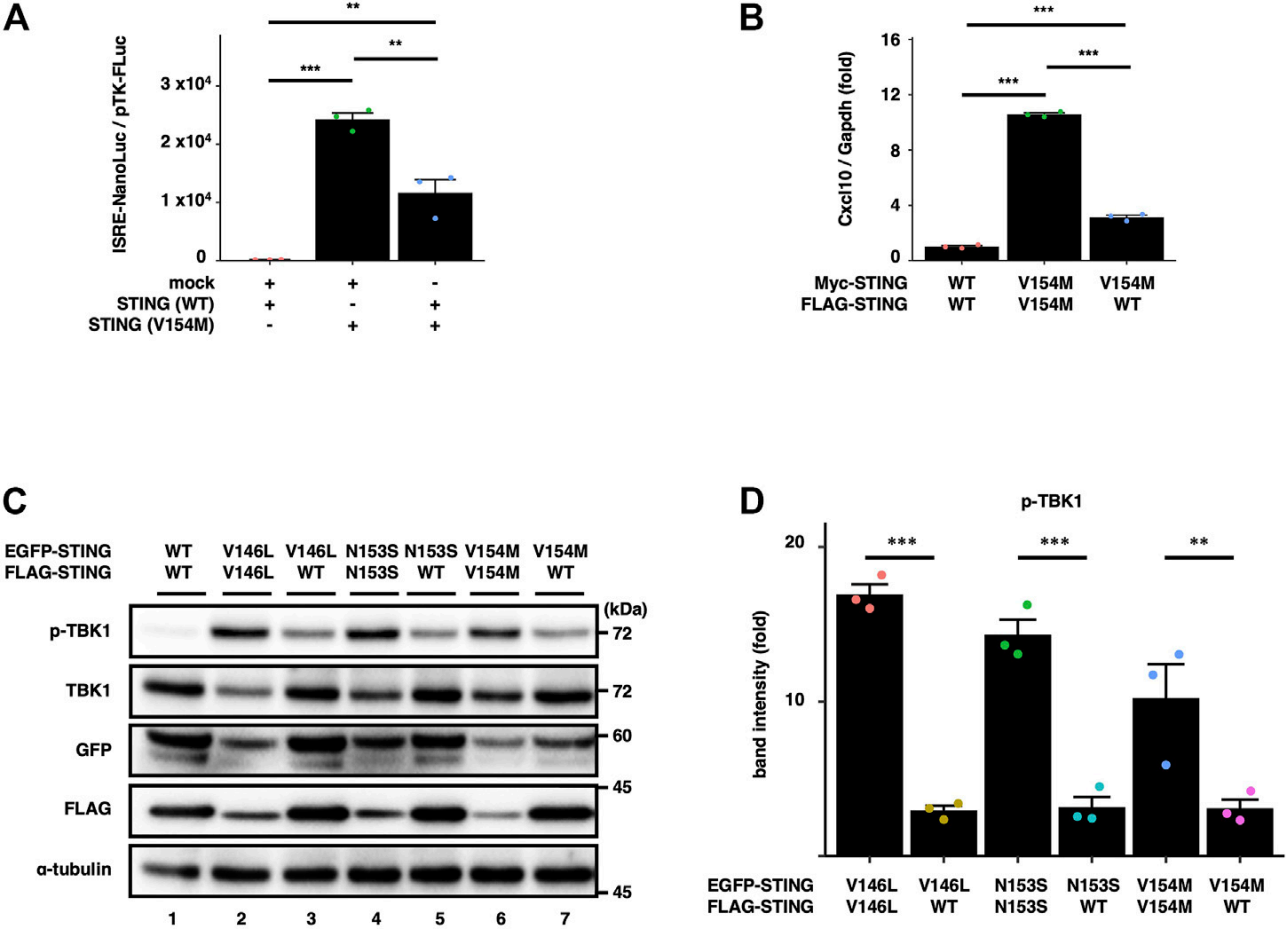
WT suppresses the SAVI-dependent STING signalling. **(A)** HEK293T cells were transfected with the plasmids encoding WT or SAVI (V154M) together with an ISRE (also known as PRDIII or IRF-E)-luciferase reporter. Luciferase activity was then measured. Data represent mean ± s.e.m. of three independent experiments. **(B)** Myc-STING and FLAG-STING were stably expressed in *Sting* ^−/−^ MEFs as indicated. The expression of *Cxcl10* was quantitated with qRT-PCR. **(C)** EGFP-STING and FLAG-STING were stably expressed in *Sting* ^−/−^ MEFs. Cell lysates were analysed by western blot. **(D)** The band intensities of p-TBK1 and TBK1 were quantified. The ratio of pTBK1/TBK1 under the indicated conditions was normalized to that of pTBK1/TBK1 in WT/WT cells (mean ± s.e.m.; *n* = 3).

To examine the effect of STING (WT) on SAVI-triggered type I interferon response, we performed a luciferase assay with HEK293T cells, which lack the expression of endogenous STING. The cells were transfected with the plasmid encoding STING (WT or V154M) and luciferase reporter with IRF3 (also known as ISRE or PRD III-I)-responsive promoter elements. The luciferase activity in the total cell lysate was then measured. The expression of STING (V154M) resulted in the enhanced type I interferon response, and the increased response was significantly suppressed by the expression of STING (WT) (Figure 1A).

We also examined the effect of the expression of STING (WT) on the activity of STING (V154M) in STING KO cells. *Sting* ^*−/−*^ mouse embryonic fibroblasts (SKO-MEFs) were reconstituted with FLAG-tagged STING (WT or V154M) and Myc-tagged STING (WT or V154M). For simplicity, we hereafter refer to STING (WT) as “WT”, STING (SAVI) as “SAVI”, and cells expressing two types of STING (WT) with different tags as “WT/WT cells”, STING (WT) and STING (SAVI) as “WT/SAVI cells”, and two types of STING (SAVI) with different tags as “SAVI/SAVI cells”. As shown (Figure 1B), the expression of an interferon-stimulated gene *Cxcl10* was significantly increased in WT/SAVI and SAVI/SAVI cells than in WT/WT cells. The expression of *Cxcl10* in WT/SAVI cells was significantly lower than that in SAVI/SAVI cells. These results suggested the inhibitory effect of WT on the activity of SAVI.

We then examined biochemically the effect of WT on the activity of SAVI. We stably expressed EGFP-tagged SAVI and FLAG-tagged WT or SAVI. As expected, phosphorylated TBK1 (p-TBK1), a hallmark of STING activation, was not detected in WT/WT cells (Figure 1C, the lane 1). In contrast, p-TBK1 emerged in WT/SAVI and SAVI/SAVI cells. Importantly, we consistently observed that the levels of p-TBK1 in WT/SAVI cells were lower than that in SAVI/SAVI cells (Figure 1D). These results suggested that WT suppressed the activity of SAVI at least at the level of TBK1 activation.

### Heterocomplex formation between SAVI and WT

**FIGURE 2:**
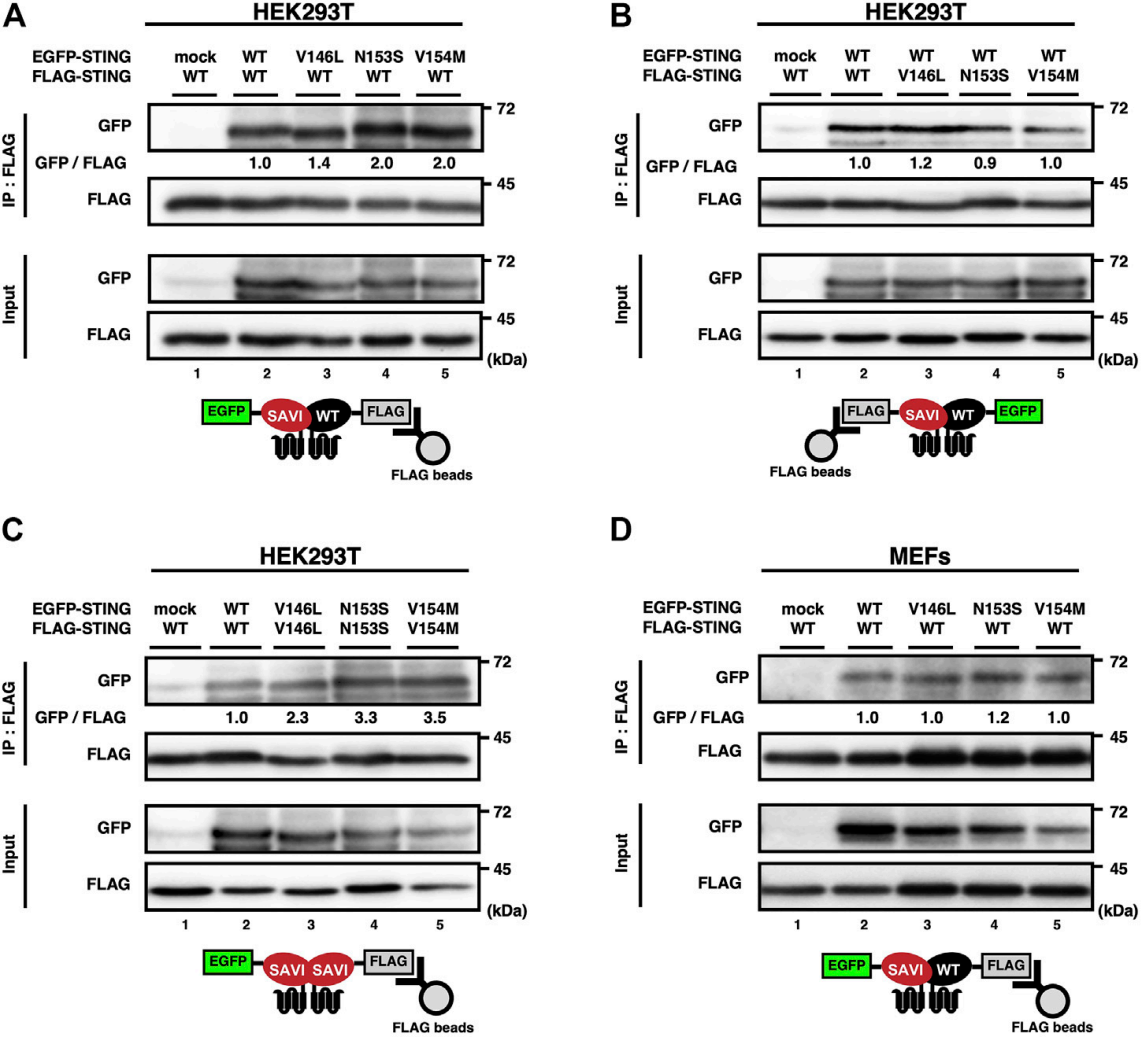
SAVI can form heterocomplex with WT. **(A–D)** HEK293T cells were transfected with the plasmids encoding EGFP-STING and FLAG-STING (**A-C**). EGFP-STING and FLAG-STING were stably expressed in *Sting* ^−/−^ MEFs (**D**). Cell lysates were prepared, and FLAG-STING was immunoprecipitated with anti-FLAG beads. The cell lysates and the immunoprecipitated proteins were analysed by western blot.

Given that WT can form a dimer or an oligomer *in vitro* (Huang et al., 2012; Ouyang et al., 2012; Shang et al., 2012; Shu et al., 2012; Yin et al., 2012; Shang et al., 2019; Zhang et al., 2019; Zhao et al., 2019; Lu et al., 2022), we reasoned that WT could also bind SAVI to exert its inhibitory function. To test the hypothesis, we analyzed the complex formation by co-immunoprecipitation, using HEK293T cells expressing EGFP-tagged WT or SAVI, and FLAG-tagged WT or SAVI. As shown (Figures 2A–C), the interaction between EGFP-WT and FLAG-WT was detected, confirming that WT could form a homocomplex without cGAMP stimulation. Intriguingly, three types of SAVIs (V146L, N153S, or V154M) bound WT, or SAVI themselves (Figures 2A–C). Similar results were obtained in SKO-MEFs stably expressing EGFP-tagged WT or SAVI, and FLAG-WT (Figure 2D). These results indicated the presence of three types of complex (WT/WT homocomplex, WT/SAVI heterocomplex, and SAVI/SAVI homocomplex) in WT/SAVI cells.

### WT/SAVI heterocomplex localizes at the endoplasmic reticulum

**FIGURE 3:**
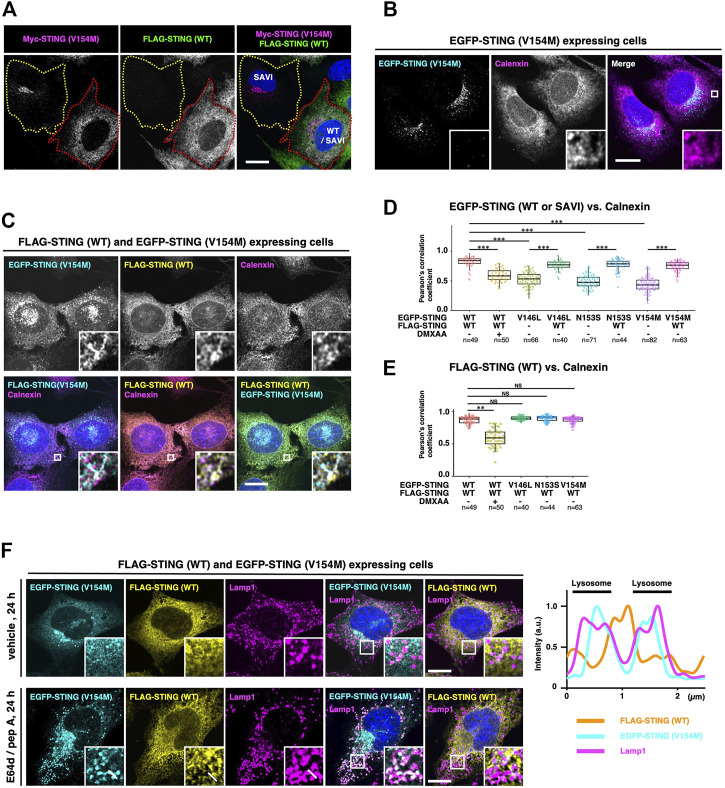
WT/SAVI heterocomplex localizes at the endoplasmic reticulum. **(A)** Myc-SAVI (V154M) alone or Myc-SAVI (V154M)/FLAG-WT was stably expressed in *Sting* ^−/−^ MEFs. Cells were mixed and plated in the same dish. Cells were then fixed and imaged by Airyscan super-resolution microscopy. The cell expressing SAVI (V154M) alone was indicated by the yellow dotted outlines. The cell expressing WT/SAVI (V154M) was indicated by the red dotted outlines. **(B)** EGFP-SAVI (V154M) was stably expressed in *Sting* ^*−/−*^ MEFs. Cells were then fixed, permeabilized, and immunostained with an anti-calnexin antibody. **(C)** EGFP-SAVI (V154M) and FLAG-WT were stably expressed in *Sting* ^−/−^ MEFs. Cells were then fixed, permeabilized, and immunostained with anti-calnexin antibody and anti-FLAG antibody. **(D)** The Pearson’s correlation coefficients between EGFP-STING and calnexin. Data are presented in box-and-whisker plots (n > 40). **(E)** The Pearson’s correlation coefficient between FLAG-WT and calnexin. Data are presented in box-and-whisker plots (n > 40). **(F)** *Sting* ^−/−^ MEFs stably expressing EGFP-SAVI (V154M) and FLAG-WT were treated with or without [E64 days (30 μg ml^−1^) and pepstatin A (40 μg ml^−1^)] for 24 h. Cells were then fixed, permeabilized, and immunostained with anti-Lamp1 antibody and anti-FLAG antibody. The fluorescence intensity profiles along the white lines are shown. Scale bars, 10 µm.

The activity of STING is tightly regulated by membrane trafficking (Taguchi and Mukai, 2019; Taguchi et al., 2021). We thus sought to examine the subcellular localizations of the hetero- and homocomplexes. Two cell lines, *i.e*., cells expressing SAVI (V154M) alone and cells expressing WT/SAVI (V154M), were plated in the same dish and examined. SAVI (V154M) localized exclusively at the perinuclear compartments in cells that do not express WT (Figure 3A, the cell indicated by yellow dotted outlines). These results indicated that SAVI (V154M) targeted the Golgi, consistently with the previous observations (Jeremiah et al., 2014; Dobbs et al., 2015; Mukai et al., 2016; Ogawa et al., 2018). In contrast, in WT/SAVI cells (Figure 3A, the cell indicated by red dotted outlines), the extensive perinuclear localization of SAVI (V154M) was lost. Thus, the expression of WT appeared to affect the localization of SAVI (V154M). Co-immunostaining experiments with calnexin or TGN38 indicated that the fraction of SAVI (V154M) localized at the ER and the TGN in WT/SAVI cells (Figure 3C and Supplementary Figure S1).

We then quantified the subcellular localization of STING with the Pearson’s correlation coefficients (PCC) between STING and an ER protein calnexin. In WT/WT cells, WT showed high PCC with calnexin, confirming that WT localized to the ER. As expected, the PCC significantly decreased after the treatment of a mouse STING agonist DMXAA (Figure 3D, Supplementary Figure S1). In cells expressing SAVI (V146L, N153S, and V154M) alone, these SAVI showed low PCC with calnexin (Figures 3B,D and Supplementary Figure S1). In contrast, the further expression of WT significantly increased PCC between SAVI and calnexin (Figures 3C,D, and Supplementary Figure S1), corroborating the results shown in Figure 3A. PCC between WT and calnexin was not affected in the presence of SAVI (V146L, N153S, and V154M) (Figures 3C,E). These results suggested that WT/WT and WT/SAVI were retained at the ER, and SAVI/SAVI translocated from the ER to the Golgi. Of note, the signals of STING and calnexin did not completely overlap in the ER (Figure 3C). This segregation may reflect the different properties of these proteins: STING constantly exits the ER and is retrieved back to the ER (Taguchi and Mukai, 2019; Taguchi et al., 2021), whereas calnexin is an ER protein that associates stably with the ER (Sasaki and Yoshida, 2019).

After its binding to cGAMP, STING sequentially moved from the ER to the Golgi to recycling endosomes and then to lysosomes for its degradation (Hu et al., 2016; Mukai et al., 2016; Gonugunta et al., 2017; Prabakaran et al., 2018; Gui et al., 2019). When STING reaches lysosomes, STING is degraded by lysosomal proteases. We exploited this nature of STING in membrane trafficking for further corroborating the subcellular localizations of the homo- and heterocomplexes. In WT/SAVI cells, both WT and SAVI (V154M) were not detected inside lysosomes in the absence of the lysosomal protease inhibitors (Figure 3F, the upper panels). In contrast, SAVI (V154M), but not WT, was detected inside lysosomes 24 h after protease inhibitors treatment (Figure 3F, the bottom panels and the fluorescent profiles). These results provided another evidence that only SAVI/SAVI constitutively exited the ER and was degraded subsequently in lysosomes, while WT/WT and WT/SAVI retained at the ER (Figure 5B).

### WT/SAVI heterocomplex is dormant

**FIGURE 4:**
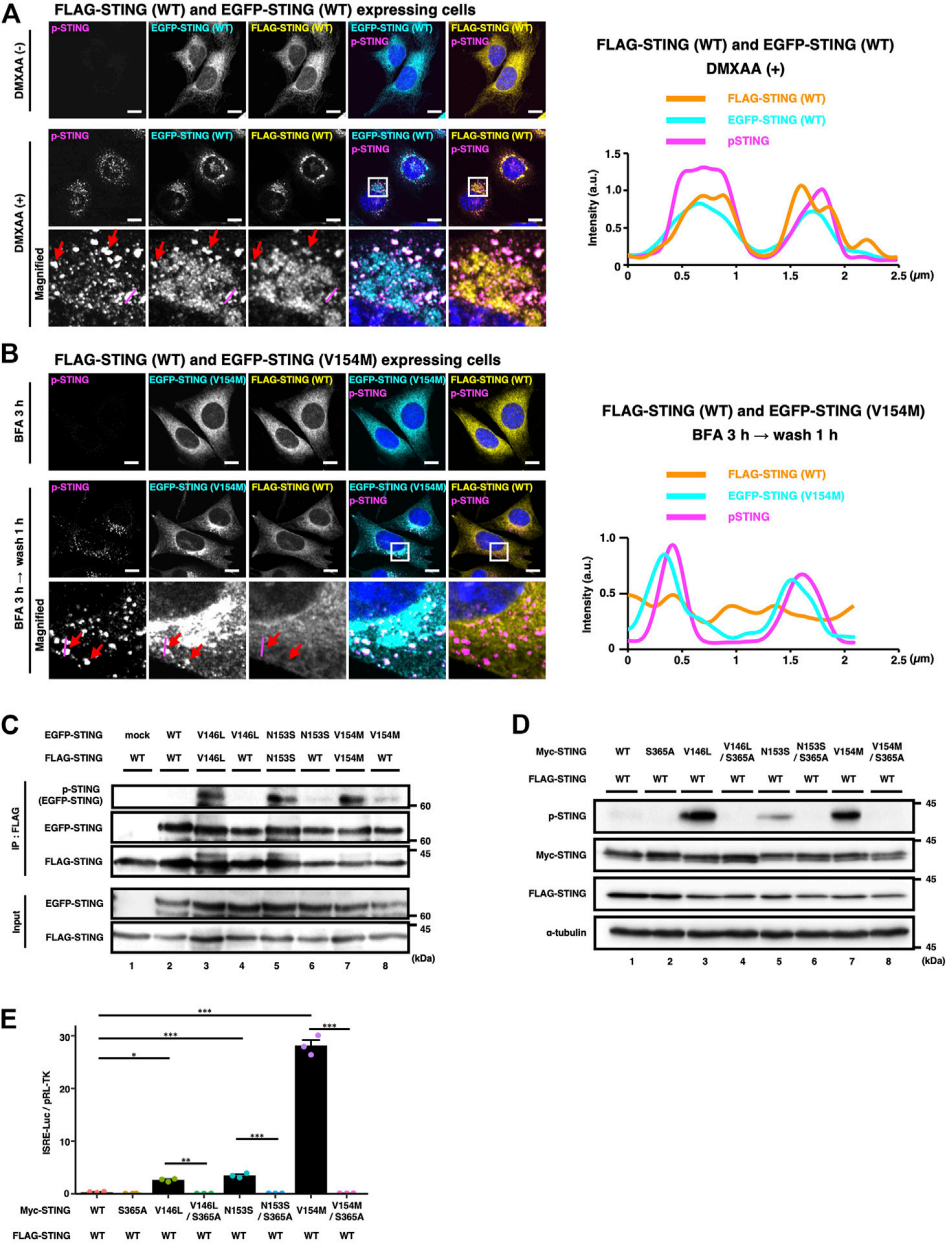
WT/SAVI heterocomplex does not activate the STING signalling. **(A)** EGFP-WT and FLAG-WT were stably expressed in *Sting* ^−/−^ MEFs. Cells were stimulated with or without DMXAA (25 μg ml^−1^) for 2 h. Cells were then fixed, permeabilized, and immunostained with anti-p-STING antibody and anti-FLAG antibody. The boxed areas are magnified in the bottom panels. The fluorescence intensity profiles along the magenta lines are shown. The red arrows indicate the puncta positive with EGFP-WT, FLAG-WT, and p-STING. **(B)** EGFP-SAVI (V154M) and FLAG-WT were stably expressed in *Sting* ^−/−^ MEFs. Cells were incubated with BFA (0.3 μg ml^−1^) for 3 h followed by 1 h incubation without BFA. Cells were then fixed, permeabilized, and immunostained with anti-p-STING antibody and anti-FLAG antibody. The boxed areas are magnified in the bottom panels. The fluorescence intensity profiles along the magenta lines are shown. The red arrows indicate the puncta positive with EGFP-SAVI (V154M) and p-STING. **(C)** HEK293T cells were transfected with the plasmids encoding EGFP-STING and FLAG-STING as indicated. Cell lysates were prepared, and FLAG-STING was immunoprecipitated with anti-FLAG beads. The cell lysates and the immunoprecipitated proteins were analysed by western blot. **(D)**. HEK293T cells were transfected with the plasmids encoding Myc-STING and FLAG-STING as indicated. Cell lysates were analysed by western blot. **(E)** HEK293T cells were transfected with the plasmids as indicated, together with an ISRE (also known as PRDIII or IRF-E)-luciferase reporter. Luciferase activity was then measured. Data represent mean ± s.e.m. of three independent experiments. Scale bars, 10 µm.

STING is suggested to act as a protein scaffold to activate TBK1 at the TGN. Activated TBK1, then, in turn, phosphorylates STING at Ser365, generating the IRF-docking site on STING (Liu et al., 2015). As expected, the signal of the phosphorylated STING (p-STING) emerged only after DMXAA treatment in WT/WT cells (Figure 4A). To characterize WT/SAVI cells by immunostaining of p-STING, we synchronized the membrane trafficking of SAVI, because SAVI is widely distributed to various organelles including the Golgi/recycling endosomes/lysosomes, making the analysis difficult (Ogawa et al., 2018). WT/SAVI (V154M) cells were treated with brefeldin A (BFA), a fungal macrocyclic lactone that blocks ER-to-Golgi traffic, and immunostained for p-STING. In the presence of BFA, WT and SAVI (V154M) localized primarily at the ER, and the p-STING signal was not detected (Figure 4B, the upper panels), consistently with the fact that SAVI requires the ER-to-the Golgi trafficking to exsert it activity. After washing out BFA, the p-STING signal then emerged at perinuclear compartments, where p-STING co-localized with SAVI (V154M), not WT (Figure 4B, the bottom panels). These results suggested that SAVI/SAVI homocomplex was active, whereas SAVI/WT heterocomplex was dormant.

To examine the activity of WT/SAVI heterocomplex in detail, we performed the co-immunoprecipitation analyses using EGFP- and FLAG-tagged STINGs, which have different molecular weights. Cell lysates of WT/SAVI cells or SAVI/SAVI cells were immunoprecipitated with anti-FLAG antibody. We then evaluated the phosphorylation of EGFP-SAVI (V146L, N153S, or V154M) that bound FLAG-WT or FLAG-SAVI (Figure 4C, Supplementary Figure S2). In SAVI/SAVI cells, the phosphorylation of EGFP-SAVI (60 kDa) was detected in the immunoprecipitates with FLAG-SAVI (Figure 4C: the lanes 3, 5, 7). In contrast, in WT/SAVI cells, the phosphorylation of EGFP-SAVI was not detected in the immunoprecipitates with FLAG-WT (Figure 4C: the lanes 4, 6, 8). Thus, these results showed that SAVI/SAVI homocomplex was active, whereas WT/SAVI heterocomplex was dormant.

At last, we examined if the activation of the STING signalling was solely dependent on SAVI in WT/SAVI cells. For this purpose, we generated SAVI variants with serine-to-alanine substitution on S365, the residue essential for the activation of IRF3. As shown (Figure 4D, Supplementary Figure S2, and Supplementary Figure S3), the signal of p-STING in WT/SAVI cells was mostly cancelled by the introduction of the S365A mutation in SAVI variants (V146L/S365A, N153S/S365A, and V154M/S365A). Furthermore, the S365A mutations into SAVI variants resulted in a significant decrease in the type I interferon response in WT/SAVI cells (Figure 4E).

### Increased binding of SAVI to Surf4 in the presence of WT

**FIGURE 5:**
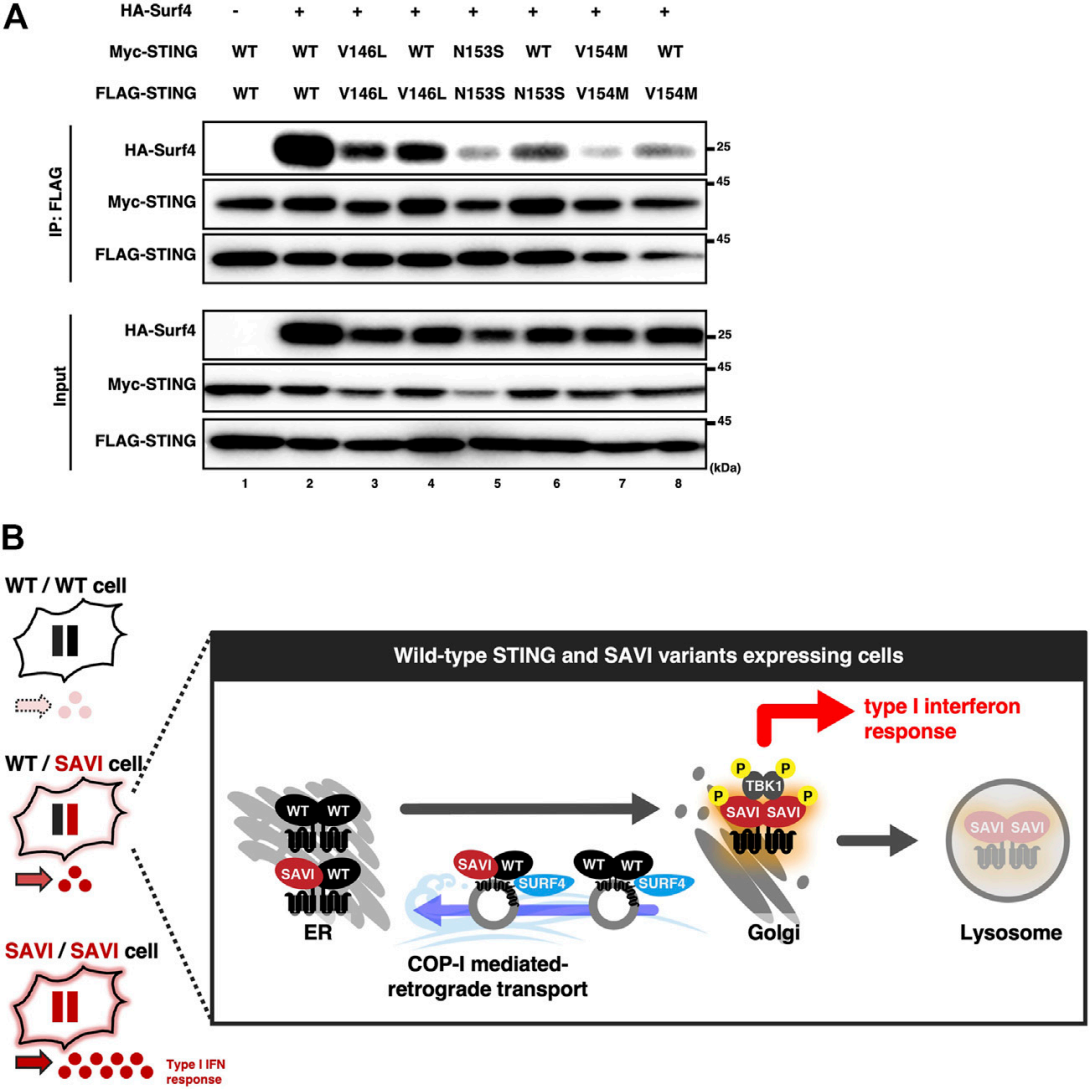
The expression of WT increased the binding between SURF4 and SAVI. **(A)** HEK293T cells were transfected with the plasmids as indicated. Cell lysates were prepared, and FLAG-STING was immunoprecipitated with anti-FLAG beads. The cell lysates and the immunoprecipitated proteins were analyzed by western blot. **(B)** A graphical abstract illustrating the three types of STING complex with their subcellular localizations.

Surf4 is a protein that circulates between the ER and the Golgi (Mitrovic et al., 2008). We and others have recently shown that STING was constantly retrieved back from the Golgi to the ER by binding to Surf4 (Deng et al., 2020; Mukai et al., 2021; Steiner et al., 2022). SAVI variants had a lower binding ability to Surf4, which may underlie the impaired localization of SAVI to the ER (Mukai et al., 2021). We thus reasoned that the binding ability to Surf4 differed between SAVI/SAVI homocomplex and WT/SAVI heterocomplex. As shown, the levels of Surf4 bound to WT were higher than that of Surf4 bound to SAVI variants (Figure 5A: lane 2 vs. lanes 3, 5, 7) (Supplementary Figure S2), confirming the previous results. Intriguingly, the levels of Surf4 bound to SAVI variants were higher in WT/SAVI cells than in SAVI/SAVI cells (Figure 5A: lane 3 vs. 4, lane 5 vs. 6, or lane 7 vs. 8). Thus, the ER localization of WT/SAVI heterocomplex may be facilitated by the interaction between Surf4 and WT present in WT/SAVI heterocomplex (Figure 5B).

## Discussion

Recent studies using SAVI mouse models revealed that homozygous SAVI embryos were not viable (Warner et al., 2017; Motwani et al., 2019). In line with the results with mouse models, SAVI patients expressing the SAVI variant (H72N, F153V, V147L, N154S, V155M, G158A, G166E, C206Y, G207E, R281Q, and R284G/S) are all heterozygous in STING alleles (Jeremiah et al., 2014; Liu et al., 2014; König et al., 2017; Melki et al., 2017; Konno et al., 2018; Saldanha et al., 2018; Keskitalo et al., 2019; Lin et al., 2021 ), except one SAVI allele (R281W) (Lin et al., 2020). Thus, WT allele appears to function dominantly over SAVI alleles in terms of viability of mouse and human. In the present study, we showed that WT could form heterocomplex with SAVI. The heterocomplex localized to the ER, and most importantly, was dormant. Given the presence of WT/WT homocomplex in WT/WT cells and that of SAVI/SAVI homocomplex in SAVI/SAVI cells (Figure 2), WT/SAVI cells are supposed to have three types of complex, *i.e.*, WT/WT homocomplex, WT/SAVI heterocomplex, and SAVI/SAVI homocomplex (Figure 5B). Among these complexes, only SAVI/SAVI homocomplex is active (Figure 4). Thus, compared to cells expressing only SAVI, WT/SAVI cells are expected to have a limited activity of the STING signalling. We propose that the dormant heterocomplex formation between WT and SAVI underlies, at least in part, the dominance of STING WT allele over SAVI alleles in the STING-triggered inflammatory response.

The severity of the symptoms in SAVI patients is highly variable (Frémond et al., 2021). The study using SAVI mouse models also showed that V154M variant had more robust STING activity than N153S variant and led to more severe disease phenotypes (Motwani et al., 2019). In the present study, WT/SAVI (V154M) showed the highest activity among three WT/SAVI heterocomplex (Figure 4E and Supplementary Figure S3), in line with the clinical manifestations. Intriguingly, we found that WT/SAVI (V154M) heterocomplex had a reduced binding ability to Surf4, compared to WT/SAVI (V146L or N153S) heterocomplex (Figure 5A). We also found that WT/SAVI (V154M) heterocomplex was preferentially associated with TGN than WT/SAVI (V146L or N153S) heterocomplex (Supplementary Figure S3). These different properties of the SAVI variants may be relevant to their different activities. Given the inhibitory effect of WT on the activity of SAVI, the expression levels of WT may also affect the severity of the SAVI symptoms.

Upon emergence of cytosolic DNA, STING translocates from the ER to the Golgi, to recycling endosomes, and then to lysosomes for its degradation (Mukai et al., 2016). The degradation of STING in lysosomes was required for the termination of type I interferon response (Gonugunta et al., 2017; Prabakaran et al., 2018; Gui et al., 2019). SAVI variants also moved from the ER to the Golgi (Ogawa et al., 2018) and to recycling endosomes (Kemmoku et al., 2022), resulting in the type I interferon responses without DNA stimulation. In the present study, we showed that SAVI/SAVI homocomplex, as WT/WT homocompelx bound to cGAMP, translocated eventually to lysosomes and degraded (Figure 3F). The transport efficiency of SAVI/SAVI homocomplex to lysosomes may be relevant to the resolution of the SAVI-triggered inflammatory signalling.

## Methods

### Antibodies

Antibodies used in this study were as follows: rabbit anti-TBK1 (ab40676, dilution 1:1000; Abcam); rabbit anti-phospho-TBK1 (D52C2, dilution 1:1000; Cell Signaling Technology); mouse anti-GFP (JL-8, dilution 1:1000; Clontech); rabbit anti-GFP (50430-2AP, dilution 1:1000; Proteintech); mouse anti-FLAG (1E6, dilution 1:1000; Wako); rabbit anti-FLAG (PM020, dilution 1:1000; MBL); mouse anti-α-tubulin (10G10, dilution 1:500; Wako); rabbit anti-calnexin (10427-2-AP, dilution 1:500; Proteintech); rat anti-lamp1 (eBio1D4B, dilution 1:5000; eBioscience); rabbit anti-phospho-STING (D1C4T, dilution 1:400; Cell Signaling Technology); sheep anti-TGN38 (AHP499G, dilution 1:200; BioRad); rabbit-phospho-STING (D8F4W dilution 1:1000; Cell Signaling Technology); rabbit anti-Myc (16286-1-AP, dilution 1:1000; Proteintech); rabbit anti-HA (C29F4, dilution 1:1000; Cell Signaling Technology); Goat anti-Rabbit IgG (H + L) Mouse/Human ads-HRP (4,050–05, dilution 1:10000) and Goat Anti-Mouse IgG (H + L) Human ads-HRP (1031–05, dilution 1:10000) (Southern Biotech); Alexa 594- or 647-conjugated secondary antibodies (A31573, A10037, A21209, A31571, A21207, A21448, dilution 1:2000; Thermo Fisher Science). For the immunoprecipitation of FLAG-tagged protein, anti-DYKDDDDK tag Antibody Beads (012–22781, Wako) were used. The antibody against STING was generated by immunizing rabbits with recombinant glutathione S-transferase (GST)-hSTING-C (amino acid 173–379) produced in *E. coli*.

### Reagent

The following reagents were purchased from the manufacturers as noted: E64 days (4321, Peptide Institute), pepstatinA (4397, Peptide Institute), DMXAA (D5235, Vadimezan), BFA (11861, Cayman).

### Cell culture

HEK293T cells were purchased from the American Type Culture Collection (ATCC). MEFs were obtained from embryos of *Sting* ^−/−^ mice at E13.5 and immortalized with SV40 Large T antigen. HEK293T and MEFs were cultured in DMEM supplemented with 10% fetal bovine serum and penicillin/streptomycin/glutamine in a 5% CO2 incubator. MEFs that stably express tagged proteins were established using retrovirus. Plat-E cells were transfected with pMXs vectors, and the medium that contains the retrovirus was collected. MEFs were incubated with the medium and then selected with puromycin (2 μg/ml) or blasticidin (5 μg/ml) for several days.

### PCR cloning

Mouse STING was amplified by PCR with complementary DNA (cDNA) derived from ICR mouse liver. The product encoding mouse STING was introduced into pMXs-IPuro or pMXs-IBla, to generate N-terminal GFP-tagged, FLAG-tagged and Myc-tagged construct. When cells stably expressing two types of STING with different tags were generated, cDNA of STING composed of synonymous codons was used to prevent homologous recombination (gBlocks Gene Fragments, Inte-grated DNA Technologies). Mouse STING was also introduced into pBABE-Puro. SAVI variants and S365A mutations into SAVI variants were generated by site-directed mutagenesis. Mouse Surf4 was amplified by PCR with cDNA derived from MEFs. The product encoding Surf4 was introduced into pMXs-IHyg-HA, to generate an N-terminal HA-tagged construct.

### Luciferase assay

HEK293T cells seeded on 96-well plates were transiently transfected with a luciferase reporter plasmid (10 ng), pTK-Fluc (1 ng) as internal control, and STING-expression plasmid in pBabe vector (20 ng). Twenty-four hours after the transfection, the luciferase activity in the total cell lysate was measured.

### qRT-PCR

Total RNA was extracted from cells and reverse-transcribed using SuperPrep^®^ Ⅱ Cell Lysis & RT Kit for qPCR (TOYOBO). Quantitative real-time PCR (qRT-PCR) was performed using KOD SYBR qPCR (TOYOBO) and LightCycler 96 (Roche). The sequences for the oligonucleotides were as follows. 5′-AGG​TCG​GTG​TGA​ACG​GAT​TTG-3’ (Gapdh; sense primer) and 5′-TGT​AGA​CCA​TGT​AGT​TGA​GGT​CA-3’ (Gapdh; antisense primer); 5′- AGT​GCT​GCC​GTC​ATT​TTC​TGC​CTC-3’ (Cxcl10; sense primer) and 5′- GCA​GGA​TAG​GCT​CGC​AGG​GAT​GAT​T-3’ (Cxcl10; antisense primer). Target gene expression was normalized based on GAPDH content.

### Immunocytochemistry

Cells were fixed with 4% paraformaldehyde (PFA) in PBS at room temperature for 15 min, permeabilized with 0.1% Triton X-100 in PBS or digitonin (50 μg/ml) in PBS at room temperature for 5min. After blocking with 3% BSA in PBS, cells were incubated with primary antibodies, then with secondary antibodies conjugated with Alexa fluorophore.

### Confocal microscopy

Confocal microscopy was performed using LSM880 with Airyscan (Zeiss) with 20 × 0.8 Plan-Apochromat dry lens, 63 × 1.4 Plan-Apochromat oil immersion lens, 100 × 1.46 alpha-Plan-Apochromat oil immersion lens. Images were analyzed and processed with Zeiss ZEN 2.3 SP1 FP3 (black, 64-bit) (ver. 14.0.21.201) and Fiji (ver. 2.0.0-rc-69/1.52p).

### Immunoprecipitation

Cells were washed with ice-cold PBS and scraped in immunoprecipitation buffer composed of 50 mM HEPES-NaOH (pH 7.2), 150 mM NaCl, 5 mM EDTA, 1% Triton X-100 or 1% CHAPS, protease inhibitor cocktail (25955, dilution) (Nacalai Tesque) and phosphatase inhibitor (8 mM Naf, 12 mM β-glycerophosphate, 1 mM Na_3_VO_4_, 1.2 mM Na_2_MoO_4_, 5 mM cantharidin, 2 mM imidazole), The cell lysates were centrifuged at 15,000 rpm for 10 min at 4°C, and the resultant supernatants were incubated for 1 h or overnight at 4°C with anti-DYKDDDDK tag Antibody Beads. The beads were washed four times with immunoprecipitation wash buffer (50 mM HEPES-NaOH (pH 7.2), 150 mM NaCl, 0.1% Triton X-100 or 0.7% CHAPS) and eluted with 2 × Laemmli sample buffer. The immunoprecipitated proteins were separated with SDS-PAGE and transferred to the PVDF membrane, then analyzed by western blot.

### Western blot

Proteins were separated in polyacrylamide gel and then transferred to polyvinylidene difluoride membranes (Millipore). These membranes were incubated with primary antibodies, followed by secondary antibodies conjugated to peroxidase. The proteins were visualized by enhanced chemiluminescence using Fusion SOLO.7S, EDGE (Vilber-Lourmat).

### Quantification of imaging data

For quantification of imaging data of multiple cells, individual cells were segmented by Cellpose, a deep learning-based segmentation method with cytosol and nucleus images. Pearson’s correlation coefficient was quantified by BIOP JACoP in Fiji plugin with ROI data from Cellpose.

### Statistical analysis

Error bars displayed throughout this study represent s.e.m. unless otherwise indicated, and were calculated from triplicate samples. In box-and-whisker plots, the box bounds the interquartile range (IQR) divided by the median, and whisker extend to maximum of 1.5 × IQR beyond the box. The corresponding data points are overlayed on the plots. Statistical significance was determined with one-way ANOVA followed by Tukey-Kramer *post hoc* test.; *p < 0.05; **p < 0.01; ***p < 0.001; NS not significant (*p* > 0.05).

## Data Availability

The original contributions presented in the study are included in the article/Supplementary Material, further inquiries can be directed to the corresponding authors.
