# Supplementary figures and images for: The activity of disease-causative STING variants can be suppressed by wild-type STING through heterocomplex formation
Source: Front Cell Dev Biol. 2022 Nov 3;10:1037999. doi: 10.3389/fcell.2022.1037999 (PMC9682468; doi:10.3389/fcell.2022.1037999)

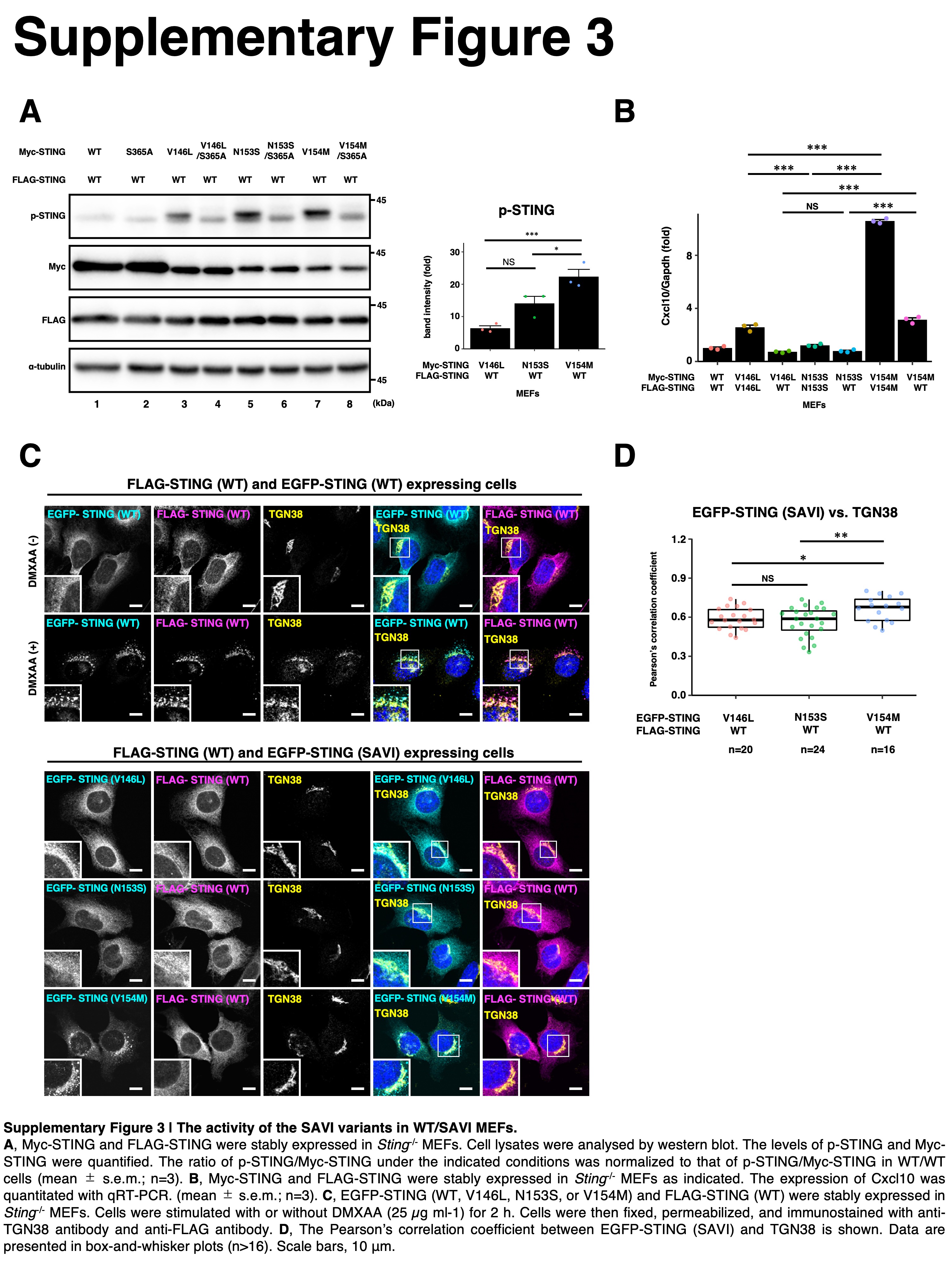

Supplement: Supplementary file 1 [file Image3.jpeg]

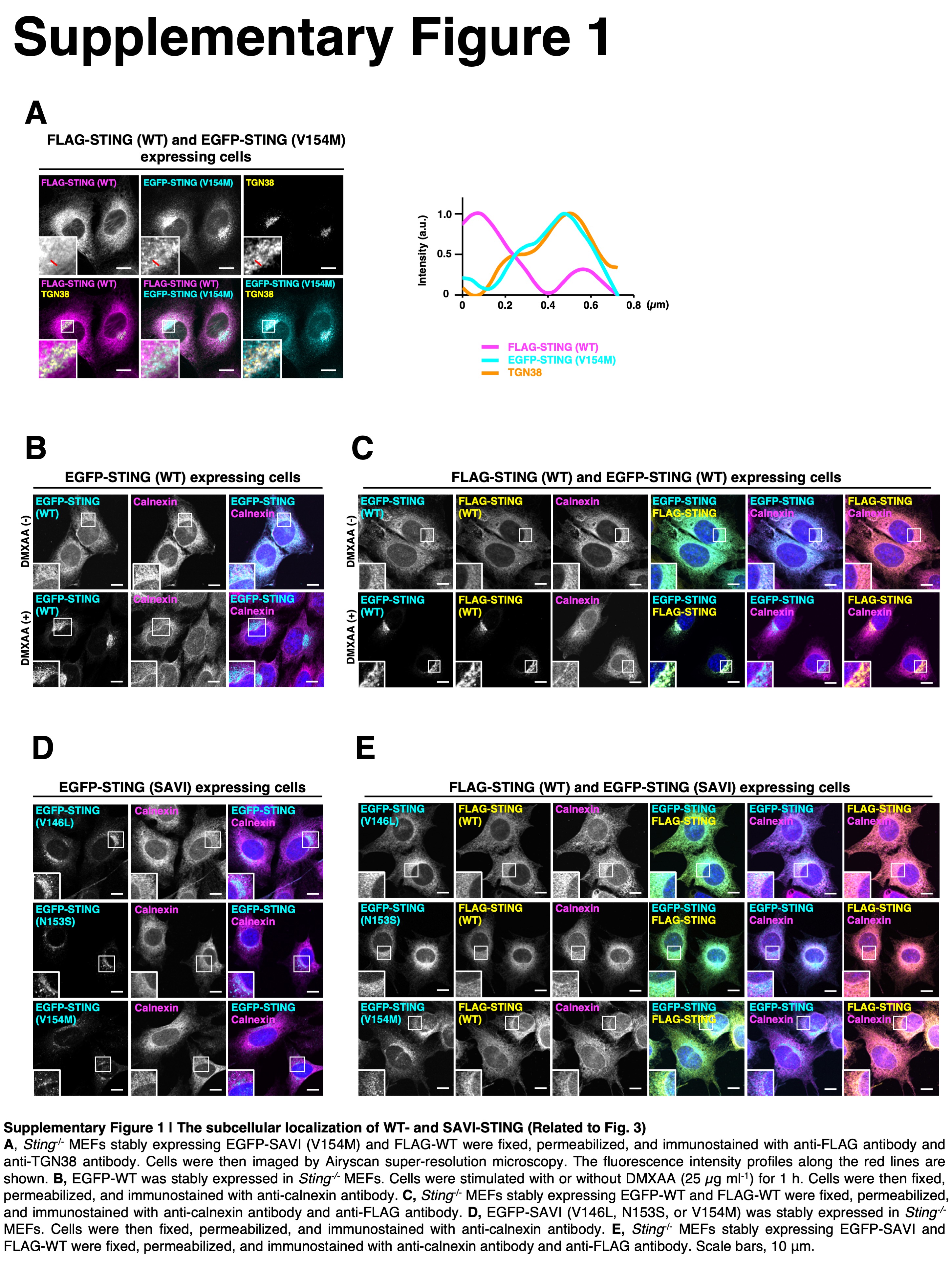

Supplement: Supplementary file 2 [file Image1.jpeg]

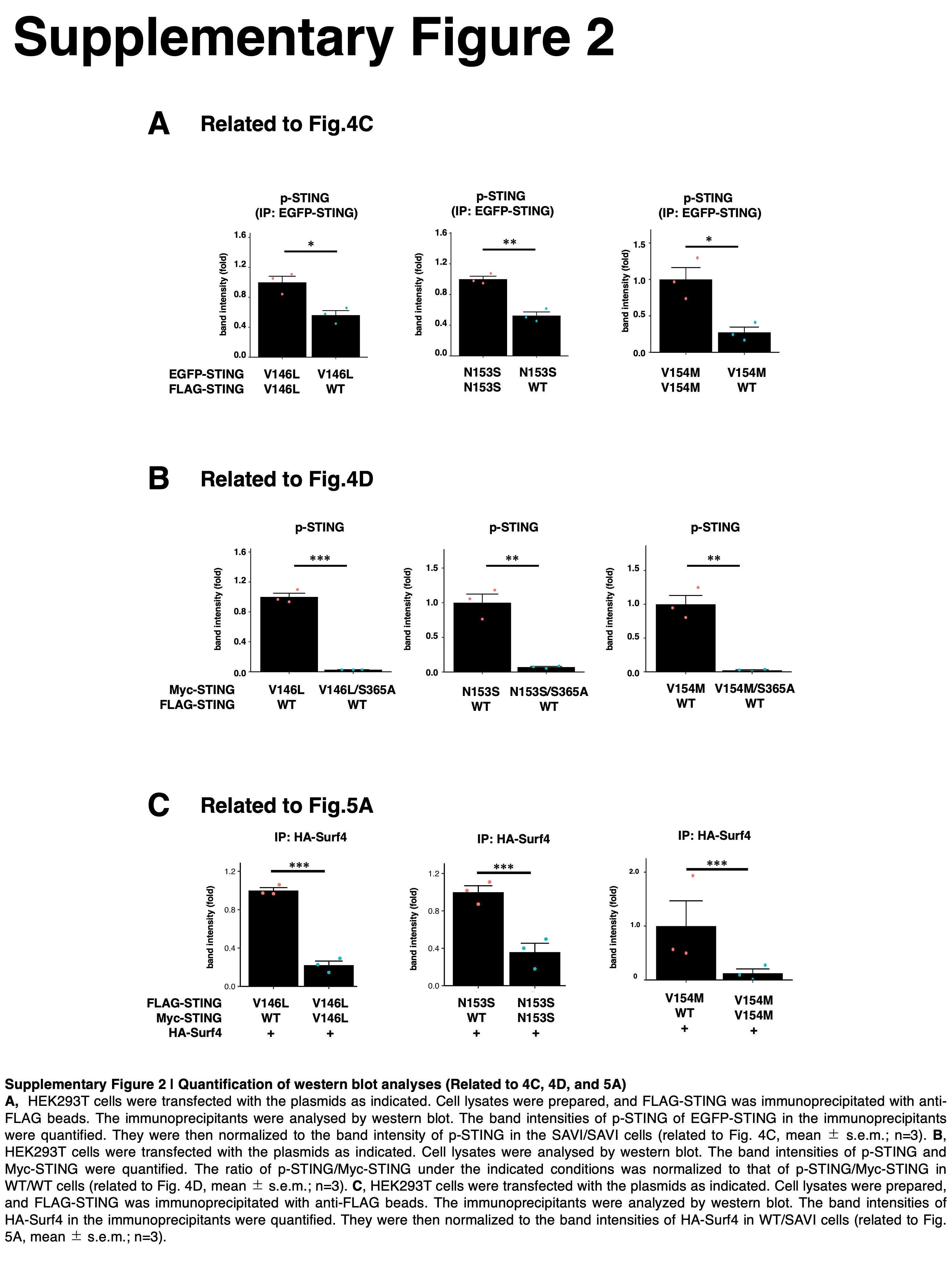

Supplement: Supplementary file 3 [file Image2.jpeg]
